# Supplementary material for: The CASPAR study protocol. Can cervical stiffness predict successful vaginal delivery after induction of labour? a feasibility, cohort study
Source: PLoS One. 2025 Jan 16;20(1):e0311324. doi: 10.1371/journal.pone.0311324 (PMC11737698; doi:10.1371/journal.pone.0311324)
Supplement: S1 Fig — (PDF) [file pone.0311324.s002.pdf]

Post-assessment questionnaire

1. Have you had a speculum before today?

   Yes No

   If yes, how many speculum examinations have you had in the past? (please circle)

   1-2 3-6 7-10 >10

2. How did you find the speculum today on a scale of 1 to 10 for discomfort? (please circle)

   1 2 3 4 5 6 7 8 9 10

   No discomfort very uncomfortable

3. The cervical stiffness test was done during the speculum examination. Did you notice it?

   Yes no not sure

   If yes, how did it feel on a scale of 1 to 10 for discomfort? (please circle a number)

   1 2 3 4 5 6 7 8 9 10

   No discomfort very uncomfortable

4. How does the cervical stiffness examination compare to having a smear? (please circle **)**
   a. better than having a smear
   b. similar to having a smear
   c. worse than having a smear
   d. I haven't had a smear

5. Did you have any concerns during the cervical stiffness test? (please comment below)

……

……

……

6. Would you agree to a speculum and cervical stiffness test again as part of future induction of labour assessment?

   Yes No not sure

   If no, please explain why not below.

   ………

   ………

   ………

7. How did you find the vaginal examination for the bishops score today on a scale of 1 to 10 for discomfort? (please circle a number)

1 2 3 4 5 6 7 8 9 10

No discomfort very uncomfortable

8. Which examination did you find most tolerable? (please circle one)

Cervical stiffness (during the speculum)	Bishops score (during the vaginal examination)	both the same

9. If we could use the cervical stiffness test results to help predict your chances for a vaginal delivery following an induction of labour, would this influence your decision to have an induction of labour? Please comment below.

……
……
……
……
……

10. Is there anything we could have done today to improve your experience for your induction of labour? Please comment below.

……
……
……
……
……

11. Is there anything else you would like to tell us about your experience of having the cervical stiffness test today? Please comment below.

……
……
……
……
……

Thankyou for your participation.

If you have any further questions regarding this study, please ask a member of the research team or email CASPAR@liverpool.ac.uk.
