# Supplementary material for: The CASPAR study protocol. Can cervical stiffness predict successful vaginal delivery after induction of labour? a feasibility, cohort study
Source: PLoS One. 2025 Jan 16;20(1):e0311324. doi: 10.1371/journal.pone.0311324 (PMC11737698; doi:10.1371/journal.pone.0311324)
Supplement: S1 File — (PDF) [file pone.0311324.s003.pdf]

Participant Consent Form

CASPAR: Can Cervical Stiffness Predict Successful Vaginal Delivery after Induction of Labour?

Name of Researchers:
Dr Elizabeth Medford, Clinical Research Fellow, University of Liverpool/ Liverpool Women's Hospital
Dr Angharad Care, Clinical Lecturer, University of Liverpool/Liverpool Women's Hospital
Dr Andrew Sharp, Senior Clinical Lecturer, University of Liverpool/Liverpool Women's Hospital

Participant Identification Number: | | | | | | |

Please put your initials in each box if you agree with the statement

1.	I confirm that I have read and understand the information sheet dated 31st July 2023 version V3.2 for the above study. I have had the opportunity to consider the information, ask questions and have had these answered satisfactorily.	☐
2.	I understand that my participation is voluntary and that I am free to leave the study at any time, without giving any reason and without my or my baby's medical care or legal rights being affected.	☐
3.	I give permission for both my own and my baby's medical notes and data collected during this research to be looked at by responsible individuals involved in this study and regulatory authorities.	☐
4.	I give permission for the researcher to contact my medical practitioner in the event of clinically significant findings from this research.	☐
5.	I give permission for cervical stiffness measurements to be taken using the Pregnolia device as part of this study.	☐
6.	I agree for a copy of this completed consent form to be sent to the Department of Women's and Children's Health, Centre for Women's Health Research, University of Liverpool (where it will be kept in a secure location), to allow confirmation that my consent for the study has been given.	☐
7.	I give permission for my data to be shared both within and outside of the UK in a fully anonymised format.	☐

Participant Consent Form

8.	I agree to take part in the above study.	☐
9.	OPTIONAL: I agree to be contacted in the future for further research related to this study.	☐

**Name of participant**	**Signature**	**Date**

**Researcher taking consent (if different to PI)**	**Signature**	**Date**

**Principal Investigator**	**Signature**	**Date**

Original to be kept by the participant; 1 copy for the researcher; 1 copy to be kept with the hospital notes
