# Supplementary material for: The CASPAR study protocol. Can cervical stiffness predict successful vaginal delivery after induction of labour? a feasibility, cohort study
Source: PLoS One. 2025 Jan 16;20(1):e0311324. doi: 10.1371/journal.pone.0311324 (PMC11737698; doi:10.1371/journal.pone.0311324)
Supplement: S2 File — (PDF) [file pone.0311324.s004.pdf]

Participant Information Sheet

CASPAR: Can Cervical Stiffness Predict Successful Vaginal Delivery after Induction of Labour?

We are inviting many of the women who attend for induction of labour to take part in a research study. Before you decide whether or not to take part it is important for you to understand why the research is being performed and what it will involve. Please take the time to read the following information carefully and discuss it with others if you wish. One of our team will go through the information sheet with you and answer any questions you may have.

Take time to decide whether or not you wish to take part.

Thank you for reading this.

Why are we doing the study?

The aim of this study is to try and find a way of predicting which women having an induction of labour will continue to have a natural birth, also known as a vaginal delivery.

Over 180,000 women a year in the UK have an induction of labour for a variety of different reasons. This process is offered to keep women and their babies safe when the option of waiting for natural onset of labour has more risks. However, there is no current way of knowing how quickly women will have their baby after an induction of labour. More importantly there is no guarantee that following induction of labour the baby will be born naturally and almost 1 out of 5 ladies who have induction of labour will require a caesarean section.

Having a test that could provide more information on how likely the induction of labour process is to lead to a natural delivery for each individual woman, and how long the labour will be, could give women and their doctors valuable information to better plan for the delivery of their baby.

Current practice in the UK before the induction of labour process can begin involves your midwife or health care professional performing a digital vaginal examination. This is to assess what is happening at the neck of the womb, also known as the cervix. From this examination a 'Bishop's Score' is calculated based on the findings of the cervix, such as how soft it feels, how long it feels and whether it is has started to open up before labour (dilate). This method has been used for over 50 years before starting an induction of labour, however recent studies have shown that calculating this score does not then tell us how successful the whole process will be and whether a natural delivery will be achieved.

New methods are being developed in research to try and improve upon the Bishop's Score assessment. One technique is a device used to measure how "stiff" the cervix is. Research using this test has already shown it to be more reliable and likely better than the Bishop's score for assessing the changes at the cervix. However, it has not yet been studied in the assessment of women prior to their induction of labour.

This study will explore whether this stiffness assessment is better at predicting who will have a natural delivery after induction of labour.

Why have I been chosen?

We are inviting all pregnant women with their first baby, aged 18 years and older who attend for induction of labour to take part in this research study.

Participant Information Sheet

Do I have to take part?

No, it is up to you to decide whether or not to take part in this study. If you decide to take part - you will be asked to sign a consent form. You will be free to withdraw from the study at any time and your routine care will not be affected. If you decide not to take part – this will not affect the care you or your family receives.

What will happen to me if I take part?

If you agree to take part in this study you will meet a member of the research team prior to your induction of labour appointment, either at the time of booking your appointment or when you attend the induction of labour suite on the day of your induction. They will provide you with verbal and written information on the research study, answer any questions you may have and ask you to provide written consent for participation.

After you have given your consent to take part, you will attend for your induction of labour as planned. You will have routine procedures prior to your induction of labour including; an ultrasound to confirm the baby is head first and monitoring of the baby's heartbeat (CTG).

The additional study procedure is called a cervical stiffness assessment. This requires you to have a speculum examination. This is the same examination as when you attend for a smear appointment. A small device will be placed on the neck of the womb during the speculum examination to gain the cervical stiffness measurement. This device creates a small vacuum to measure how soft the cervix is. Three measurements will be taken during the same assessment and the whole procedure should take no longer than five minutes. This will be performed by an experienced practitioner.

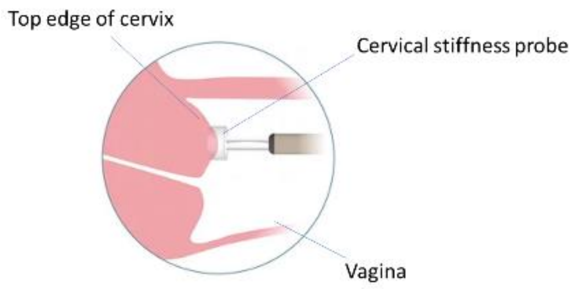

Following this you will continue with your induction of labour as planned where a member of the midwifery induction of labour team will undertake a digital vaginal assessment and determine if you require any medication into the vagina as part of the routine induction process.

After you have had both assessments, the study cervical stiffness assessment and the routine digital vaginal assessment, we will ask you to complete a short questionnaire outlining your experience of both assessment techniques.

After your baby is born, we will collect information from your electronic medical records and your baby's electronic medical records regarding the events during and after your delivery. You will not be directly contacted at this time.

Will my taking part in this study be kept confidential?

Yes. We will follow ethical and legal practice and all information will be handled in confidence. Any information you give us will be used by the research team in the course of the research. All data will be stored securely and no personal data will be available to the researchers. However, if any results provide clinically relevant information, we will inform your medical doctor. If there are any incidental findings during the cervical examination that require follow up, we will inform your GP.

If we were to share the information collected during this study with other researchers, hospitals, universities, non-profit institutions and commercial companies in the UK, it would be done so in a fully anonymised format (meaning those receiving the information will not be able to identify you). We will share fully anonymised findings from this study with the cervical stiffness device manufacturer, Pregnolia, in Switzerland.

Participant Information Sheet

How will you use my data?

We (study sponsor – the University of Liverpool) will need to use information from you and from your medical records for this research project.

This information will include your initials, name, date of birth, NHS number and contact details (address, telephone number and email). People will use this information to do the research or to check your records to make sure that the research is being done properly.

People who do not need to know who you are will not be able to see your name or contact details. Your data will have a code number instead and therefore be fully anonymised.

We will keep all information about you safe and secure. It will be stored for 15 years before sensitive disposal.

Once we have finished the study, we will keep some of the data so we can check the results. We will write our reports in a way that no-one can work out that you took part in the study.

What are my choices about how my information is used?

- You can stop being part of the study at any time, without giving a reason, but we will keep information about you that we already have.
- We need to manage your records in specific ways for the research to be reliable. This means that we won't be able to let you see or change the data we hold about you.
- If you agree to take part in this study, you will have the option to take part in future research using your data saved from this study.

Where can I find out more about how my information is used?
You can find out more about how we use your information

- at www.hra.nhs.uk/information-about-patients/
- our leaflet available from www.hra.nhs.uk/patientdataandresearch
- by asking one of the research team
- by sending an email to legal@liverpool.ac.uk, or
- by ringing us on 0151 795 0523.

What are the possible benefits of taking part?

The results of this research will not be available in the course of your pregnancy and delivery and will not directly benefit you. However, we hope that the results of the study will enable us to improve future antenatal care provided to women and inform their management surrounding induction of labour and mode of delivery. The results of this study could help with decisions in your next pregnancy.

What are the possible risks of taking part?

The risks involved in this study have been carefully assessed and the main objective of the research team is to maintain yours and your baby's safety at all time. The additional study procedure to be done, called cervical stiffness assessment, requires you to have a speculum examination and a small device placed on the cervix for a few moments. A speculum is the same examination as when you attend for a smear appointment. Some women may find this uncomfortable, but it has no risk to you or your baby. This will be performed by an experienced practitioner.

Taking part in this research will add on time to your induction of labour assessment, however no additional visits to the hospital will be required.

Participant Information Sheet

What will happen if I don't want to continue in the study?

You are free to withdraw at any time throughout the course of the study, without explanation. The care you or your family receives will not be affected in anyway. If you withdraw from the study, we will not perform any additional study investigations on you.

What will happen to the results of the research study?

It is intended that once the study is complete the results will be published as research papers in medical journals. No data will be published that will allow individuals to be identified.

We will also engage with our patient support groups via social media to share our findings. Please visit "Liverpool Babies" on facebook or twitter, or get in touch at liverpoolbabies@liverpool.ac.uk.

Where can I get further information or discuss any problems?

Please visit https://en.pregnolia.com/ for further information regarding the cervical stiffness measurement device including patient experience testimonials and videos of the device in use.

If you have any questions or worries about any aspect of this study, please contact a member of the delivery suite team on …………………… If your concerns are not resolved, you can contact the Patient Advisory Liaison Services (PALS) on 0151 702 4353. You can also visit PALS by asking at the hospital reception.

Who is organising and funding the research?

The University of Liverpool is the Sponsor of this research and is conducting this research study. The University of Liverpool holds indemnity and insurance cover with Newline Insurance Company, which will apply to this study. The study is funded by The Harris-Wellbeing Preterm Birth Centre and is managed by the Centre for Women's Health Research, University of Liverpool.

Who has reviewed the study?

All research in the NHS is looked at by an independent group of people, called a Research Ethics Committee, to protect your interests. This study has been reviewed for ethical considerations and given a favourable opinion by members of the Seasonal Research Ethics Committee.

Contact for further information

Should you have any further queries regarding this study, please contact:

Dr Elizabeth Medford, Clinical Research Fellow, Harris Wellbeing Preterm Birth Centre, Centre for Women's Health Research, The University of Liverpool, Liverpool Women's Hospital, Crown Street, Liverpool, L8 7SS

Email: CASPAR@liverpool.ac.uk

Thank you for taking the time to read and consider this information sheet. Should you decide to take part in the study, you will be given a copy of the information sheet and a signed consent form to keep.
