# Supplementary material for: The CASPAR study protocol. Can cervical stiffness predict successful vaginal delivery after induction of labour? a feasibility, cohort study
Source: PLoS One. 2025 Jan 16;20(1):e0311324. doi: 10.1371/journal.pone.0311324 (PMC11737698; doi:10.1371/journal.pone.0311324)
Supplement: S3 File — (DOCX) [file pone.0311324.s005.docx]

**CASPAR**

**Can Cervical Stiffness Predict Successful Vaginal Delivery After Induction of Labour?**

Version 2.2

17/8/24

MAIN SPONSOR: University of Liverpool

FUNDERS: Harris- Wellbeing Preterm Birth Centre and the Centre for Women’s Health Research

REC reference: 23/LO/0627

IRAS ID: 316356

**Study Team**

Chief Investigator: Dr Andy Sharp

Principal Investigator: Dr Elizabeth Medford

Co-Investigator: Dr Angharad Care

Statistician: Steven Lane

**Study Coordination Co-ordinator/Contact for Clinical Queries**

For general queries, supply of Study documentation, and collection of data, please contact: Study Coordinator: Dr Elizabeth Medford

Address: Harris Wellbeing Preterm Birth Centre

Centre for Women’s Health Research

The University of Liverpool

Liverpool Women’s Hospital

Liverpool

L8 7SS

Email: [Elizabeth.medford1@lwh.nhs.uk](mailto:Elizabeth.medford1@lwh.nhs.uk)

**STUDY SUMMARY**

| **Title** | Can cervical stiffness predict successful vaginal delivery after induction of labour? |
| --- | --- |
| **Design** | A prospective, observational, feasibility study of 100 women with singleton pregnancy undergoing induction of labour.  Women will undergo cervical stiffness assessment through the aspiration technique, prior to digital vaginal examination for Bishop’s score assessment, at the time of attendance for planned induction of labour. Women will then undergo routine induction of labour as per the unit policy; either prostaglandins, Cook’s catheter or artificial rupture of membranes with potential oxytocin use.  Data related to outcomes and variables will be collected from the patient’s hospital notes and electronic computer records after delivery. Data analysis will be undertaken to determine if cervical stiffness assessment can be used as a predictive tool for the outcome of vaginal delivery after induction of labour. |
| **Aims** | To determine if cervical stiffness assessment using the Pregnolia System can be used to predict successful vaginal delivery after induction of labour and how it compares with the Bishop’s score assessment. |
| **Objectives** | - To inform for the design of an appropriately powered study to assess the capability of this novel device for IOL prediction. - To explore the acceptability of the cervical stiffness assessment in patients undergoing IOL. - To obtain cervical stiffness measurements in primiparous women prior to term IOL to:   - Determine the reliability and best interpretation of triplicate measurements in this patient group.   - Explore any potential association between cervical stiffness assessment and vaginal delivery following IOL.   - Compare to BS assessments taken concurrently and explore the association with outcome of IOL. |
| **Outcome Measures** | Feasibility outcomes; recruitment rate, participant acceptability of cervical stiffness assessment at IOL, adherence to protocol and data collection, cervical stiffness assessment fidelity.  Primary clinical outcome will be vaginal delivery. |
| **Population Eligibility** | Inclusion criteria:   - Age ≥ 18 years - Being induced - Singleton pregnancy - Primiparous - ≥37+0 weeks gestation - Intact membranes - Able to provide informed consent   Exclusion criteria:   - Previous cervical surgery - Any cervical pathology at 12 o’clock position on cervix - Vaginal bleeding evident on examination - Visible, symptomatic cervical or vaginal infections - Known congenital uterine anomalies - Known or suspected structural/chromosomal fetal abnormality - Known HIV - Cervical carcinoma |
| **Duration** | 12 months recruitment. |

**Patient & Public Involvement Group**

Patient and public involvement (PPI) has been an essential aspect in the development of CASPAR. We have received input and feedback from Liverpool Babies PPI group. It was agreed that the research question was of importance and the design of the study would be acceptable to women undergoing an induction of labour. In addition, we collaborated with the PPI group in creating our lay summary and study materials including a patient information sheet. Throughout the study the PPI group will provide a forum for patient support and help disseminate results of study findings.

**KEYWORDS**

Induction of labour, cervical stiffness, cervical integrity

**Study Flow Chart**
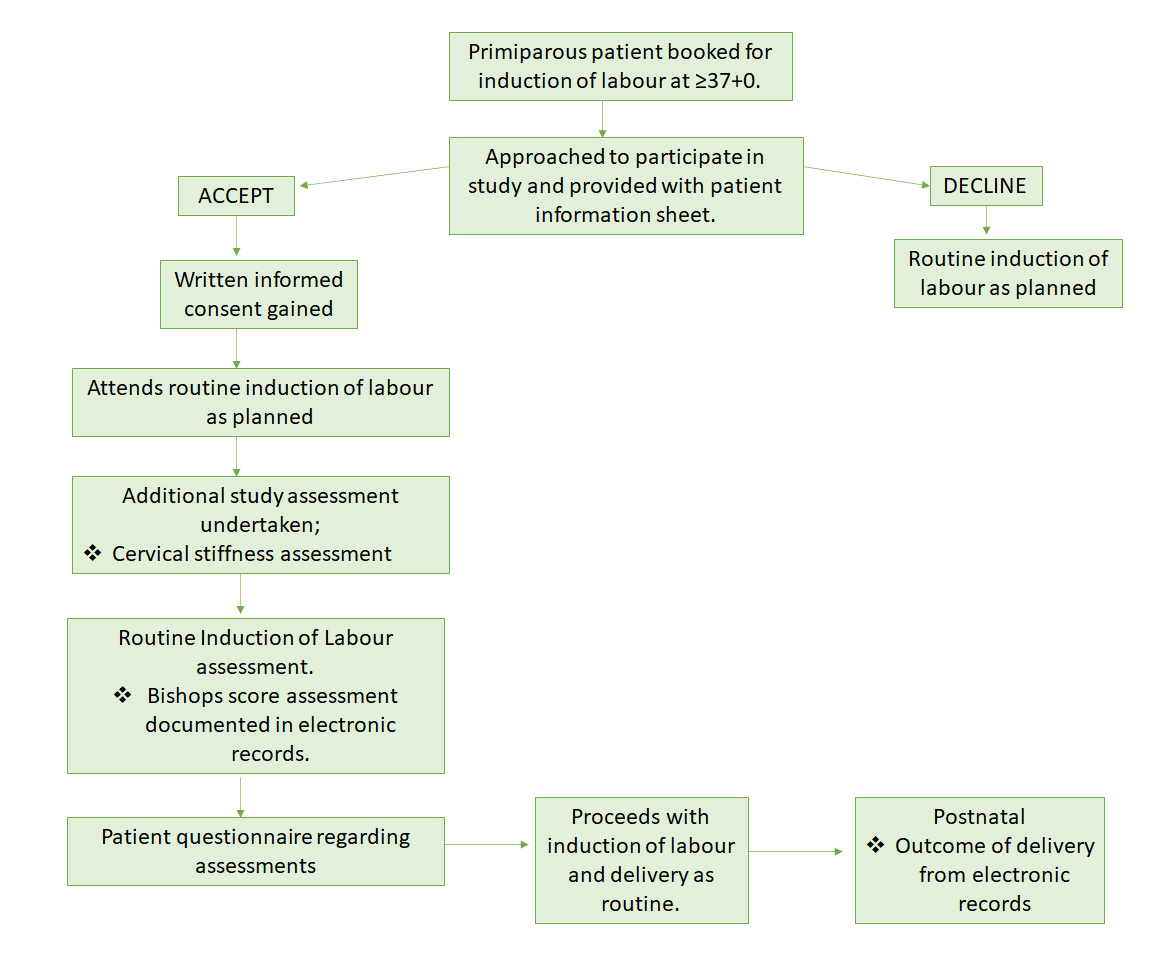


**Glossary of Abbreviations**

**AE** *Adverse Event*

**CCI** *Cervical Consistency Index*

**CI** *Chief Investigator*

**CRF** *Case Report Form*

**CSI** *Cervical Stiffness Index*

**CTG** *Cardiotocography*

**FFN** *Fetal Fibronectin*

**GCP** *Good Clinical Practice*

**GDPR** *General Data Protection Regulation*

**HIE** *Hypoxic Ischaemic Encephalopathy*

**HRA** *Health Research Authority*

**HWPBC** *Harris Wellbeing Preterm Birth Centre*

**ICF** *Informed Consent Form*

**ICH** *International Conference on Harmonisation*

**ISF** *Investigator Site File*

**LWH** *Liverpool Women’s Hospital*

**NHS** *National Health Service*

**NICE** *National Institute for Health and Care Excellence*

**NICU** *Neonatal Intensive Care Unit*

**PI** *Principal Investigator*

**PIN** *Patient Identification Number*

**PIS** *Patient Information Sheet*

**PPI**  *Patient and Public Involvement*

**QC** *Quality Control*

**REC** *Research Ethics Committee*

**ROC** *Receiver Operating Characteristic*

**SAE** *Serious Adverse Event*

**SD** *Standard Deviation*

**SMG** *Study Management Group*

**TVUSS** *Transvaginal Ultrasound*

**UoL** *University of Liverpool*

Table of Contents

| **1. INTRODUCTION**  1.1 Background  1.2 Rationale for current study | 11  11  14 |
| --- | --- |
| **2. STUDY OBJECTIVES** | 14 |
| **3. STUDY DESIGN**  3.1 Study procedures  3.2 Study outcome measures  3.3 Loss to follow up  3.4 Study Closure | 14  15  16  17  17 |
| **4. PARTICIPANT ENTRY AND RECRUITMENT**  4.1 Eligibility criteria  4.2 Recruitment | 17  17  19 |
| **5. ADVERSE EVENTS**  5.1 Definitions  5.2 Reporting Procedures  5.3 Notes on grading of adverse events  5.4 Relationship to study procedures  5.5 Expectedness  5.6 Reporting | 20  20  21  21  22  22  23 |
| **6. STATISTICS AND DATA ANALYSIS**  6.1 Introduction  6.2 Sample size  6.3 Outcome measures  6.4 Interim Analysis and Monitoring  6.5 Statistical Methodology | 24  24  25  25  25  25 |
| **7. REGULATORY ISSUES**  7.1 Ethics approval  7.2 Consent  7.3 Confidentiality  7.4 Quality assurance and quality control of data  7.5 Records retentions  7.6 Study discontinuation  7.7 Indemnity  7.8 Sponsor  7.9 Funding  7.10 Audits | 27  27  27  27  28  28  29  29  29  29  29 |
| **8. STUDY MANAGEMENT**  8.1 Study Management Group  8.2 Oversight Committees | 29  29  30 |
| **9. END OF STUDY** | 30 |
| **10. MONITORING**  10.1 Risk Assessment  10.2 Source Data  10.3 Data Capture Methods  10.4 Monitoring Methods  10.5 Clinical Site Monitoring | 30  30  31  31  32  35 |
| **11. ARCHIVING** | 36 |
| **12. PUBLICATION POLICY** | 36 |
| **13. REFERENCES** | 37 |
| **14. APPENDICES**  14.1 Required documentation  14.2 Schedule of study procedures | 39  39  39 |

**1. INTRODUCTION**

This protocol describes the CASPAR Study and provides information about procedures for entering participants, study procedures, safety reporting and governance requirements. Every care was taken in its drafting, but corrections or amendments may be necessary. These will be circulated to investigators in the Study. Problems relating to this study should be referred, in the first instance, to the Chief Investigator (CI).

This study will adhere to the principles outlined in the UK Policy Framework for Health and Social Care Research. It will be conducted in compliance with the protocol, the Data Protection Act 2018 and the UK General Data Protection Regulation (GDPR) as amended from time to time and any successor legalisation in the UK and any other directly applicable regulation relating to data protection and privacy as well as any other regulatory requirements as appropriate.

- 1. **BACKGROUND**

Induction of labour is a common medical intervention in obstetrics with a gradually rising trend over the last decade (1). In England in 2020-21 34% of deliveries were induced compared to 21% in 2010-11. With over 550,000 deliveries in England in 2020-21, clinical practice surrounding induction of labour impacts upon over 180,000 women each year in England alone(2). Global trends are similar with induction of labour rates now reaching over 30% in Australia and the United States of America and 35% in Sri Lanka (3-5).

Induction of labour is indicated for a variety of clinical reasons, but overall is considered when the outcome for the baby, mother or both will be better if achieving a more imminent vaginal delivery compared to awaiting the spontaneous onset of labour (5). However, induction does not come without risks. It is known to be associated with an increased rate of assisted vaginal delivery, increased epidural rate and can impact upon on the birth experience of the woman (1, 6). More importantly, almost 20% of inductions do not result in a vaginal birth and require an unplanned caesarean section which in itself has additional maternal and neonatal risks, as well as an increase in cost and use of healthcare resources (7, 8). A reliable prediction model to determine which women will successfully achieve a vaginal delivery following induction of labour will allow more informed, shared decision making regarding the risks of induction against the likelihood of achieving a vaginal delivery, taking into consideration the indication for induction and its timeframe.

At present there is no reliable predictive bedside tool for determining who will have a successful vaginal delivery following induction of labour (7). Since the 1960s the initial assessment of a woman prior to induction of labour is a digital vaginal examination to assess the cervix and calculate the Bishop’s score. This examination is routine practice throughout the UK still today and can be performed by any trained medical professional. The Bishop’s score describes five characteristics of the cervix: dilatation, effacement or cervical length, position, consistency of the cervix and station of the presenting part, calculating up to a maximum score of 13. The higher the score, the more favourable the cervix and a successful induction of labour is expected (9). In the original description of this assessment by Bishop for multiparous women, it was determined that a score >9 would have no failure in induction of labour, and the average duration of labour is less than 4 hours (10). Since then, further studies have elected that a modified Bishop’s score of greater than 6 will indicate a successful vaginal birth within 6hrs in 90% of cases (11, 12). However more recently, digital palpation of the cervix through the Bishop’s score has been shown to have wide inter-observer and intra-observer variability and is not a sufficiently reproducible nor reliable method of assessment due to its subjective nature (13, 14). Overall, the Bishop’s score has now been recognised as a poor predictor of the outcome of labour induction(15, 16).

Newer techniques for pre-induction assessment of the cervix have been explored including transvaginal ultrasound (TVUSS) cervical length and chemicals related to pregnancy such as fetal fibronectin and insulin-like growth factor. In comparison to the Bishop’s score they have shown equivalence and not improved predictability of the outcome of induction, as well as requiring more expensive and labour intensive resources (12).

TVUSS assessment can objectively measure the cervical length, as well as give additional information such as evidence of cervical funnelling and the angle of the cervix. These have been shown to have their own predictive value in success of vaginal birth after induction and cannot be elicited from a Bishop’s score assessment (9, 17, 18). However, studies directly comparing the performance of TVUSS cervical assessment and the Bishop’s score in the predictor of outcome of induction of labour have given mixed and inconsistent results. The 2015 Cochrane review into methods for assessing pre-induction cervical ripening concluded that there is insufficient evidence to support the use of TVUSS over the standard digital vaginal assessment and that outcomes of vaginal birth or caesarean section were the same in both groups (9). Again, a recent comparative clinical trial concluded that TVUSS cervical length measurements predicts the success of induction of labour with similar diagnostic accuracy to the conventional Bishop’s Score (14).

Fetal fibronectin (FFN) is a glycoprotein found in the amniotic fluid and is known to leak into the vaginal secretions prior to the onset of spontaneous labour. When used as a predictive marker in the induction of labour setting, there was an association between having both a negative FFN and a low Bishop’s score and a higher risk of prolonged labour and need for operative delivery. However, studies overall have shown no additional benefit to FFN over Bishop’s score in predictive outcome (9).

Recent attention has turned to the potential of assessing the biomechanical properties of the cervix such as cervical consistency and elasticity (19). A systematic review showed initial promise of cervix sonoelastography as a predictive tool for successful vaginal delivery after medication induction of labour, and was determined to be more reliable than the Bishop’s score (20). This had led to the development of a new cervical consistency index (CCI) score using TVUSS to assess the dynamic properties of the cervix as pressure is applied. When the tissue is softer the CCI is lower, therefore CCI correlates negatively with gestational age (19). In preterm birth studies, use of CCI scores have proven to be effective in the prediction of spontaneous preterm birth (21, 22). However, when this association was transferred for assessment in the induction of labour setting there was no correlation found between CCI and the mode of delivery after induction of labour. Both the vaginal delivery group and caesarean section group after induction of labour had similar mean CCI scores (19).

Cervical elasticity can be assessed through a novel technique of aspiration using a vacuum assisted device, called the Pregnolia system. The device tip is applied to the anterior lip of the cervix and gives a cervical stiffness index (CSI) score. In a pilot study comparing CSI between 50 non-pregnant and 50 pregnant women results showed quantitative assessment of cervical softening during pregnancy was detectable by the aspiration technique and there is progressive cervical softening during pregnancy with recovery of cervical stiffness to pre-pregnancy levels in the postpartum period (23). This study has suggested the potential use of the Pregnolia system as an objective assessment of the cervix for use in the induction of labour setting and outcome prediction.

A further study has compared the Pregnolia system assessment and digital palpation assessment of silicone cervix models to ascertain any inter- and intra-observer variability. The results clearly demonstrated that digital palpation was an unreliable method to assess cervical stiffness. In comparison, the Pregnolia system showed a repeatable and reproducible objective method to assess cervical stiffness with superiority over digital palpation (13). However, the value of the Pregnolia system assessment shown in this study cannot be directly compared to the Bishop’s score assessment used in induction of labour prediction due to the use of silicone cervix models that don’t represent all the qualities of a native cervix other than cervical stiffness.

The aspiration technique for cervical stiffness has been studied in the setting of misoprostol administration prior to intrauterine contraception insertion. This study showed the aspiration technique was able to detect pharmacologically induced cervical changes following misoprostol use and confirms misoprostol has a detectable softening effect on cervical tissue (24). These findings could propose further research in the induction of labour setting and demonstrates potential of cervical stiffness assessment in confirming cervical changes from prostaglandins commonly used as induction of labour agents. This could provide the basis of an induction of labour outcome prediction model after the use of prostaglandins using cervical stiffness assessments.

- 1. **RATIONALE FOR CURRENT STUDY**

In current practice there is no evidence-based reliable tool for assessment in predicting the outcome of induction of labour. Despite being proven to be subjective, unreliable and a poor predictor of induction of labour outcome, the Bishop’s score assessment is still the widely accepted assessment prior to induction of labour used in maternity services across the world.

Development of an effective predictive assessment for induction of labour outcome has the potential to transform maternity service planning, as well as provide women and clinicians with more robust information to allow more informed decisions surrounding care at the time of induction of labour.

Preliminary studies into the Pregnolia system have shown potential scope for objective cervical assessment of the cervix through cervical stiffness assessment and ongoing prediction of the outcome of induction. CASPAR will build upon these studies and undertake aspiration technique cervical stiffness assessments on women prior to their induction of labour, compare this to the current gold standard Bishop’s score assessment and aim to correlate these assessments with the outcome of labour.

**2. STUDY OBJECTIVES**

The CASPAR study has the following objectives;

1. To inform for the design of an appropriately powered study to assess the capability of this novel device for IOL prediction.
2. To explore the acceptability of the cervical stiffness assessment in patients undergoing IOL.
3. To obtain cervical stiffness measurements in primiparous women prior to term IOL to:
   1. Determine the reliability and best interpretation of triplicate measurements in this patient group.
   2. Explore any potential association between cervical stiffness assessment and vaginal delivery following IOL.
   3. Compare to BS assessments taken concurrently and explore the association with outcome of IOL.

**3. STUDY DESIGN**

We will conduct a single site prospective, feasibility study of 100 women with singleton pregnancy undergoing induction of labour at the Liverpool Women’s Hospital (LWH).

Participants will be recruited directly from the induction of labour suite at the time of attendance for planned induction of Labour. If they meet all the eligibility criteria, clinical staff will inform a member of the research team who will attend and approach the patient for recruitment to the study. As per NICE guidelines they will have routine procedures prior to induction of labour including; confirmation of cephalic presentation and a CTG to confirm normal fetal heart rate and absence of uterine activity.

The potential participants will be given verbal information on the study, a patient information sheet (PIS) to read and the opportunity to ask further questions. If the woman agrees to participate, they will sign the study specific informed consent form (ICF) and be registered onto a bespoke electronic data capture system that will generate a unique participant identification number (PIN).

Women will be asked to undergo a sterile speculum examination for cervical stiffness assessment, prior to routine vaginal digital examination for Bishop’s score assessment. Women will then undergo routine induction of labour as per the unit policy, either prostaglandins, or placement of Cook’s catheter (Bishop’s score <6) or transfer to delivery suite for artificial rupture of membranes and possible commencement of oxytocin infusion (Bishop’s score ≥ 6). All study involvement will take place at the time of routine induction of labour proceedings to limit additional time taken and inconvenience to the participant.

Participants will be asked to complete a short questionnaire regarding their experience of the cervical stiffness assessment and the Bishop’s score assessment.

Once recruited the participants will remain in the study until after delivery and discharge from hospital for the mother and baby, or 1 month after delivery, whichever comes first. We will collect routine clinical data from all participants’ notes and electronic hospital records for maternal and neonatal outcomes.

**3.1 STUDY PROCEDURES**

**3.1.1 Cervical Stiffness measurement**

Firstly, cervical stiffness will be measured via sterile speculum examination. The cervical stiffness is assessed by using the Pregnolia System, composed of an active component, the Pregnolia Control Unit and a disposable probe, the Pregnolia Probe. The device will be used according to its instructions for use. Briefly, the measurement location is inspected for any measurement contraindication (see 4.1.3 exclusion criteria) and excessive mucous is removed with a swab from the cervical surface. Next, the single-use, sterile Pregnolia Probe is placed on the anterior lip of the cervix at 12 o’clock position and a recording of cervical stiffness is generated over maximum 60 seconds (typically ̴15 seconds) and recorded as Cervical Stiffness Index (CSI) in mbar. The measurement is repeated 3 consecutive times without any time lag.

This procedure will be undertaken by a member of the research team trained in speculum examination and trained to use the Pregnolia system.

**3.1.2 Bishop’s Score assessment**

Secondly, routine Bishop’s score assessment will be undertaken by a sterile digital vaginal examination. The study site uses an electronic maternity notes software called K2 and the Bishop’s score is documented in a standard format. Five components of the vaginal examination will be documented as a score; cervical dilatation (cm), consistency of cervix, cervical canal length (cm), position of cervix and station of presenting part in relation to ischial spines, giving a potential score from 0 to 12.

This procedure will be undertaken by a member of the induction suite midwifery team as per routine unit practice, and their score will be documented in the patient’s electronic maternity records.

**3.2 STUDY OUTCOME MEASURES**

**Feasibility Outcomes**

Our feasibility outcomes of interest relate to whether the study procedure is acceptable, participant recruitment is achieved, data collection is feasible, and cervical stiffness assessment fidelity is maintained with adequate reliability and safety(25).

Our feasibility outcomes will be defined as follows;

1. Recruitment rate
   1. Measured as proportion of participants recruited compared to total number approached for recruitment in the study period
   2. Reasons for non-participation collected
2. Participant acceptability of cervical stiffness assessment at IOL
   1. Qualitative questionnaire following assessment
   2. Number of participant withdrawals throughout the study duration
3. Adherence to protocol and data collection
   1. Measured as number of protocol deviations
   2. Missing data
4. Cervical stiffness fidelity
   1. Ability to obtain triplicate cervical stiffness measurements
   2. Triplicate measurement reliability

**Clinical Outcomes**

Clinical outcome data will be collected in accordance with the “short-term” core outcome set for trials on IOL as determined by the international Delphi study (26, 27). Ability to capture this core data set in this feasibility study will inform data collection and study design for a larger definitive IOL study.

The primary clinical outcome will be vaginal delivery. Secondary maternal and neonatal outcomes will be recorded for descriptive analyses. Variables that are already recognised as informative for induction prediction modelling will be collected and included for descriptive analyses such as; maternal demographics, maternal obstetric parameters, induction methods and indications, cervical findings on digital examination and fetal parameters(7, 28).

**3.3 LOSS TO FOLLOW UP**

We do not anticipate any loss to follow up as the study outcome is so short.

**3.4 STUDY CLOSURE**

Study enrolment at LWH will be stopped when the total requested number of participants for the study have been recruited. The study management group (SMG) may recommend that the study be stopped prematurely. In such circumstances of premature termination / suspension of the study, the National Research Ethics Committee (REC) and Health Research Authority (HRA) will be notified according to the standard reporting guidelines.

**4. PARTICIPANT ENTRY AND RECRUITMENT**

**4.1 ELIGIBILITY CRITERIA**

**4.1.1 Pre-registration Evaluations**

All participants will require confirmation of cephalic presentation by ultrasound and a normal CTG with absence of uterine activity before recruitment to the study.

**4.1.2 Inclusion Criteria**

- Age ≥ 18 years
- Being induced
- Singleton pregnancy
- Primiparous
- ≥37+0 weeks gestation
- Intact membranes
- Able to provide informed consent

**4.1.3 Exclusion Criteria**

- Previous cervical surgery
- Any cervical pathology at 12 o’clock position on cervix
- Vaginal bleeding evident on examination
- Visible, symptomatic cervical or vaginal infections
- Known congenital uterine anomalies
- Known or suspected structural/chromosomal fetal abnormality
- Known HIV
- Cervical carcinoma

**4.1.4 Withdrawal Criteria**

We do not have formal stop criteria for the CASPAR study. However, we will establish a SMG which will meet at least monthly and will have the authority to recommend a halt to recruitment should there be concerns about patient safety.

In consenting to take part in the study, participants are consented to a speculum examination for cervical stiffness assessment and associated data collection and follow-up on pregnancy outcomes. If voluntary withdrawal occurs the participant should be given appropriate care until discharge.

The critical data in CASPAR study is derived from cervical stiffness measurement. Although participants can refuse examination at any time, refusal to have cervical stiffness measurements result in significant compromise to the study. Therefore, any participant who does not have cervical stiffness measurements would need to be withdrawn from the study. During the study procedure prior to cervical stiffness measurement, the cervix is inspected for any previously unknown cervical pathology at 12 o’clock position, new vaginal bleeding or visible cervical or vaginal infections. If any of these are present, the assessment cannot be undertaken based on manufacturer guidelines for use of the medical device. The patient would then be withdrawn from the study based on cervical criteria.

Foreseeable reasons where a participant may withdraw from the study generally apply to the additional need for a speculum examination and collection of cervical stiffness measurements and include:

- Participant withdraws consent,
- Newly diagnosed cervical criteria at time of study procedure
- Loss of capacity during the study, and
- Any other change in the participant’s condition that justifies the discontinuation in the clinician’s opinion.

A participant is free to withdraw from the study at any time. In addition, the CI may decide, for reasons of medical prudence, to withdraw a participant. In either event, the Sponsor will be notified and the date and reason(s) for the withdrawal will be documented in the participant source data. If a participant withdraws or is withdrawn, ideally, they should remain in the study for the collection outcome data. Generally, follow-up will continue unless the participant explicitly also withdraws consent for follow-up.

Participants who withdraw from the study for other reasons will be given routine standard care until delivery and will be asked to allow analysis of already anonymised data and collection of pregnancy outcome data. If the participant explicitly states their wish not to contribute further data to the study, collected data will be removed from the study database.

**4.1.5 Patient Transfers**

This is not anticipated during the course of this study

**4.1.6 Stopping Criteria**

There are no formal stopping criteria for this study. The need to stop the study will be determined by the SMG and the decision will be based upon data integrity and participant safety.

**4.1.7 COVID precautions**

Liverpool Women’s NHS Foundation Trust is well prepared to deal with the impact of COVID-19 and as such has adopted a number of measures that are intended to keep visitors and staff safe. These include performing temperature checks at the entrance to the hospital, asking staff and visitors to use antibacterial hand gel and wear medical face masks that are provided at the door.

In addition, all study participants will be asked if they are experiencing any symptoms of potential COVID-19 infection, such as persistent cough, loss of taste or sense of smell or a fever/increased temperature. Staff at LWH are required to wear medical facemasks, disposable aprons and gloves whilst they care for study participants, regardless of the COVID-19 swab result.

**4.2 RECRUITMENT**

**4.2.1 Participant selection**

The majority of participants will be recruited directly from the induction of labour suite when they attend for their planned induction of labour. We will also endeavor to recruit patients when the induction of labour is booked during an attendance in antenatal clinic, during an inpatient admission, or attendance to the maternity assessment unit. At the time of identification, a member of the core clinical research team on site will be contacted and will attend to discuss their potential participation. Participants will be given written and verbal information on the CASPAR study, as well as an opportunity ask questions and take any additional time required to consider taking part in the study.

All patients that are identified as eligible and are then approached for the study will be included in the recruitment rate. Those that agree to participate will go on to enrolment and registration (see 4.2.3).

**4.2.2 Informed Consent**

On meeting a member of the research team, potential participants will be provided with an additional PIS to refer to, the opportunity to ask questions, discuss study objectives, risks and inconveniences of the study and the conditions under which the study is to be conducted. More time will be offered for consideration of participation if necessary and it will be made clear to the participant that the quality of medical care will not be adversely affected if they decline to participate in this study and they may withdraw from the study at any time.

Once a participant is happy to consent, they will be asked to sign the study-specific Informed Consent Form (ICF) in the presence of a member of the research team. Three copies of the consent form will be collected, the original given to the participant, one placed within the participants hospital notes and one held at the Centre for Women’s Health Research (CfWHR). The principal investigator (PI) will be required to review and sign all consent forms.

The PI will ensure that all members of the research team obtaining consent comply with applicable regulatory requirements, adhering to GCP and to the ethical principles that have their origin in the Declaration of Helsinki. Members of the research team will also have received study specific training relating to eligibility screening and the informed consent / registration process and CV’s will be reviewed of all team members to ensure adequate experience in gaining consent in a research capacity.

The PIS sent to potential participants has been reviewed by our Liverpool Babies PPI group who assisted on layout, language and formulation of lay explanations to make it easily understood. In addition, previous PIS and consent forms used in singleton preterm birth studies at the LWH were reviewed to see what format of information is acceptable for participating women.

**4.2.3 Enrolment and registration**

Once eligibility has been confirmed by the PI at site the participant will be registered onto the study using an electronic registration platform. Although it will be recommended that participants take a minimum of 24 hours to consider taking part in this study, the provision for allowing participant consent within a shorter period-of-time will be permitted- this is to reduce the number of examinations the patient undergoes, ensuring the cervical stiffness assessment will be done at the same time as the routine digital vaginal examination prior to commencement of the induction of labour. Documentation of reasons for non-inclusion will be detailed in the site screening logs and forwarded to the Research Manager at the CfWHR.

Once a participant consents to taking part in the study, they will be registered on a bespoke electronic data capture system that will generate a unique participant identification number (PIN).

**5. ADVERSE EVENTS**

**5.1 DEFINITIONS**

**5.1.1 Adverse Event (AE)**

An AE is defined as any untoward medical occurrence (i.e., any unfavourable or unintended sign including abnormal laboratory results, symptom or disease) in a research participant taking part in a clinical research study.

**5.1.2 Serious Adverse Event (SAE)**

An SAE is defined as any untoward and unexpected medical occurrence or effect that

• results in death,

• is life threatening – refers to an event in which the participant was at risk of death at the time of the event. It does not refer to an event which hypothetically might have caused death if it were more severe,

• requires hospitalisation or prolongation of existing inpatient hospitalisation,

• results in persistent or significant disability or incapacity

• Is a congenital anomaly or birth defect, and

• Is otherwise considered medically significant by the investigator

Medical judgement should be exercised in deciding whether an AE is serious in other situations. Important AEs that are not immediately life‐threatening or do not result in death or hospitalisation but may jeopardise the participant or may require intervention to prevent one of the other outcomes listed in the definition above, should also be considered serious.

**5.2 REPORTING PROCEDURES**

All AEs for this study will be recorded at each study visit on the study case report form (CRF). This study does not require the formal reporting of any non-serious AEs. This is a non-interventional study. We therefore do not anticipate a large number of SAEs.

Investigators must report all SAEs from enrolment to the end of the study. In addition, maternal death and maternal life-threatening complications, stillbirths and neonatal deaths are also pre-specified outcomes to be reported immediately as SAEs. All deaths reported as SAEs as part of this study will be reviewed regularly by the SMG and Sponsor. Urgent reporting of any SAEs in relation to the study medical device or study procedures are required. All SAEs must be reported within 24 hours of sites becoming aware of the event. Reporting should be completed by downloading the XXX study SAE form from the study file repository within REDCap, completing all fields on the form and then send it to the Study Co-Ordinator at the CfWHR. All SAE’s will be subject to regular review by the SMG.

**5.3 NOTES ON GRADING OF ADVERSE EVENTS**

The assignment of the severity/grading should be made by the investigator responsible for the care of the participant using the definitions below. Regardless of the classification of an AE as serious or not. Its severity must be assessed according to medical criteria alone using the following categories.

**Table 1. Grading of Adverse Events**

| **Grading** | **Criteria/Guidelines** |
| --- | --- |
| **Mild** | Does not interfere with routine activities (awareness of symptoms or signs, but easily tolerated [acceptable]). |
| **Moderate** | Interferes to some extent with routine activities (enough discomfort to interfere with usual activity [disturbing]) |
| **Severe** | Impossible to perform routine activities (incapacity to work or to do usual activities [unacceptable]) |
| **Life Threatening** | Results in risk of death, organ damage, or permanent disability (unacceptable) |
| **Death** | Results in death (unacceptable) |

A distinction is drawn between serious and severe AEs. Grading is a measure of intensity (see above) whereas seriousness is defined using the criteria in section 8.1 hence, a severe AE need not necessarily be a SAE.

**5.4 RELATIONSHIP TO STUDY PROCEDURES**

The assignment of the causality / relatedness should be made by the investigator responsible for the care of the participant using the definition in table 3 below. If any doubt about the causality / relatedness exists the local investigator should notify the CI. In the case of discrepant views of causality between the investigator and other, the Sponsor, HRA and REC will be informed of both points of view within the regulatory reporting timeframes.

**Table 2. Definition of Relatedness**

| **Relationship** | **Description** |
| --- | --- |
| **None** | There is no evidence of any causal relationship. N.B. An alternative cause for the AE should be given. |
| **Unlikely** | There is little evidence to suggest there is a causal relationship (e.g. the event did not occur within a reasonable time after administration of the study intervention). There is another reasonable explanation for the event (e.g. the participant’s clinical condition, other concomitant treatment). |
| **Possibly** | There is some evidence to suggest a causal relationship (e.g. because the event occurs within a reasonable time after the study intervention). However, the influence of other factors may have contributed to the event (e.g. the participant’s clinical condition, other concomitant treatments). |
| **Probably** | There is evidence to suggest a causal relationship and the influence of other factors is unlikely. |
| **Highly Probable** | There is clear evidence to suggest a causal relationship and other possible contributing factors can be ruled out |

**5.5 EXPECTEDNESS**

Expectedness should be assessed by the PI at site and the CI determining as to whether the SAE is in direct response to the study procedures.

**5.6 REPORTING**

Any questions concerning AE reporting should be directed to the Study Co-Ordinator in the first instance.

**5.6.1 Non Serious Adverse Events**

All AEs should be reported on the study CRF at the time of assessment (please see study schedule).

**5.6.2 Guidance on Reporting Serious Adverse Events**

The CASPAR SAE form can be downloaded from the study management portal. This should be completed by the Investigator at site. The Investigator should assess the SAE in terms of severity, causality / relatedness and expectedness. They should assess expectedness as to whether the SAE is in direct response to study procedures.

In the absence of the PI, the SAE form should be completed by a designated member of the site clinical research team (as detailed on the site delegation log). The responsible individual should complete a final check of the SAE form, ensuring that all fields are complete and accurate – including no personal information relating to the study participant. Once this is confirmed, they should sign and date the form and forward to the CASPAR Study coordinator at the CfWHR. Immediate reporting of SAE forms can be via:

1. email to the CASPAR secure email account at TBC, or

2. Fax to CASPAR Study Co-Ordinator, CASPAR Management Team, Centre for Women’s Health Research, Department of Women’s and Children’s Health, University of Liverpool 0151 795 9599.

For urgent safety queries and where both fax and email systems have failed, please contact the CASPAR Study Co-Ordinator on (TBC)

An acknowledgement for all SAE reports will be sent to the site on the same day as the receipt of the report (where receipt is Monday – Friday, 9:00 – 17:00. For out of hours, weekends and bank holidays an acknowledgement will be sent by 11:00 on the next working day. Please note – if no acknowledgement is received by the site within the timeframes set out here, they should contact the Centre for Women’s Health Research on 0151 795 9565 to confirm the SAE report has been received.

Following the completion of the SAE form and forwarding this to the Study Management team (as set out above); the PI must then notify their relevant R&D Department of the event, as per their standard local procedure.

In the event that an SAE requires follow-up, this should confirm that recovery is complete and the participant has returned to normal or stabilised. Follow-up information should be provided on the same CASPAR SAE form Additional supporting copies of test results can be provided separately.

The Investigator at site should ensure that the participant must be identified only by their unique study identification number and date of birth. The participants’ personal information must not be included on any correspondence.

All investigators must ensure that multiple SAEs are reported separately on different forms - one report should be provided for each overall diagnosis.

Reports of related or unexpected SAEs should be submitted to the Sponsor and REC by the Study Co-Ordinator within 15 days of the CI and Study Co-Ordinator becoming aware of the event. This report should be completed on the NRES SAE form for non-IMP studies.

**5.6.3 Additional Reporting**

All SAEs will be reported to Sponsor, REC and the HRA in line with regulatory requirements. Line listings will be provided to PIs and Sponsor on a three-monthly basis: these will also be provided to the SMG. All SAEs will be reviewed as part of central monitoring by the SMG.

**6. STATISTICS AND DATA ANALYSIS**

**6.1 INTRODUCTION**

In the following section a brief overview of planned analyses for this study will be set out.

Once informed consent has been gained for participation in CASPAR participants will be registered onto the study database and a unique PIN will be generated. Participants will be registered onto the study database in accordance with the study registration operating procedures and registration will be performed by authorised CASPAR research staff using an electronic system. This system will be available 7 days per week, 24 hours a day and will be accessed by delegated site staff using a secure password protected website. All research staff will receive comprehensive training on the use of this system, which will be documented and stored in the Investigator Site File (ISF). In the unlikely event that the back-up registration process is activated, participant registration will be performed by the Study Co-Ordinator.

Data from the CASPAR CRFs will be entered onto a bespoke study database with extensive data validation checks alerting all missing data to be queried. Missing data will be monitored and strategies will be developed to minimise its occurrence. Central statistical data monitoring will summarise missing or inconsistent data periodically. The study CRF will be approved by the CI and validations will also be made that will cross check the study CRF with the CASPAR study database.

**6.2 SAMPLE SIZE**

LWH has 14 available induction of labour appointments available per day for both inpatient and outpatient inductions. We anticipate at least 50% of patients attending to meet eligibility for this study. We hope to achieve a recruitment rate of 50% from this patient group. Over a 12-month period, we would therefore conservatively expect to recruit 100 women.

As this is an exploratory piece of work a formal sample size calculation has not been performed.

**6.3 OUTCOME MEASURES**

Feasibility outcomes will be collected as outlined in section 3.2

The primary clinical outcome will be vaginal delivery.

Secondary maternal and neonatal outcomes will be recorded for descriptive analyses:

- Maternal outcomes; induction to delivery interval, induction agent(s), category of caesarean section if undertaken, indication for caesarean section if undertaken, oxytocin use, tocolytic use, epidural analgesia use, estimated blood loss, obstetric anal sphincter injury, instrumental delivery
- Neonatal outcomes; stillbirth or neonatal death, apgar score <7 at 5 minutes, NICU admission, infant requiring cooling, HIE grade 3 or more, cord PH at delivery

In order to evaluate the associations between cervical stiffness and the main outcome in the presence of other variables, we will capture the following data from electronic medical records; maternal age, body mass index at booking, ethnicity, neonatal weight, neonatal gender, gestational age at induction, indication for induction of labour.

**6.4 INTERIM ANALYSIS AND MONITORING**

As there are no formal hypotheses being tested, there are no formal stopping rules (other than safety) or mechanisms defined here to stop the study prior to the planned end of study. The study with have a study management group that will be able to review at regular intervals all accumulating data, with the responsibility of reviewing the recruitment of participants, the collection of all essential data and to assess participant safety.

**6.5 STATISTICAL METHODOLOGY**

Descriptive statistics will be generated and presented as means (SD), median (IQR) and frequency of observations (percentages) with 95% confidence intervals as appropriate.

Reliability assessment of the Pregnolia System using Cronbach’s alpha and descriptive statistics with 95% confidence intervals will inform best use of the triplicate CSI measurements. Specifically exploring whether the first, average, median or lowest measurement of the three readings should be utilised in further analysis and most importantly inform best use of the CSI results in clinical practice.

Diagnostic performance of cervical stiffness assessments using the Pregnolia System for IOL outcome will be demonstrated through receiver operating characteristic curves with area under the curve and 95% confidence intervals being calculated, as well as aiming to define the optimum cut-off value for predicting vaginal delivery.

Multivariate analysis will be performed using logistic regression, including CSI and other variables related to IOL outcome, such as maternal age, gestational age and maternal weight at booking(28). Results will be presented as odds ratios with 95% confidence intervals.

**6.5.1 Participant Groups for Analyses**

Entire observational cohort of 100 women with singleton pregnancies undergoing induction of labour.

**6.5.2 Significance levels**

As this is an exploratory study no formal levels of significance are set. All statistics presented will be presented alongside 95% confidence intervals so as to give an indication of the level of precision only.

**6.5.3 Missing data**

The likelihood of missing data is small given the standard procedure in place to manage the study centrally. Final analyses will take place on a complete-case basis with no adjustments made (e.g. multiple imputation) in the case of missing data.

**6.5.4 Exposure to Intervention**

This is a non-interventional study.

**6.5.5 Trigger for Final Data Analyses**

Analysis of study data will take place once all participant data and samples have been received and processed; with corresponding laboratory data available for analysis.

**6.5.6 Data Descriptors**

Continuous data will be summarised as median, inter-quartile range and ranges. Categorical data shall be summarised as frequencies of counts and associated percentages.

**7. REGULATORY ISSUES**

**7.1 ETHICS APPROVAL**

Before the start of the study, a favourable opinion will be sought from the UK Health Departments Research Ethics Service NHS REC and the Health Research Authority for the study protocol, informed consent forms and other relevant documents.

The study will be submitted to the proposed research site for Confirmation of Capacity and Capability. The study will be conducted in accordance with the recommendations for physicians involved in research on human subjects adopted by the 18th World Medical Assembly, Helsinki 1964 and later revisions

**7.2 CONSENT**

Consent to enter the study must be sought from each participant only after a full explanation has been given, an information leaflet offered and time allowed for consideration. Signed participant consent should be obtained. The right of the participant to refuse to participate without giving reasons must be respected. After the participant has entered the study the clinician remains free to give alternative treatment to that specified in the protocol at any stage if he/she feels it is in the participant’s best interest, but the reasons for doing so should be recorded. In these cases the participants remain within the study for the purposes of follow‐up and data analysis. All participants are free to withdraw at any time from the protocol treatment without giving reasons and without prejudicing further treatment.

**7.3 CONFIDENTIALITY**

The Chief Investigator will preserve the confidentiality of participants taking part in the study and will abide by the Data Protection Act 2018 and the UK GDPR as amended from time to time and any successor legislation in the UK and any other directly applicable regulation relating to data protection and privacy. All members of the research team will have received ICH GCP training and are thus aware of the importance of patient confidentiality. Individual participant medical information obtained as a result of this study is considered confidential and disclosure to third parties is prohibited.

At time of consent participants will be allocated a unique participant identification number. A key to this information along with consent forms (containing patient identifiers) and pseudonymised CRFs (identified by participant identification number only) will be stored separately in locked filing cabinets, located in secure rooms within the CfWHR, UoL, with restricted keycard access to nominated clinical research/admin staff who work directly for the unit. Data from pseudonymised CRFs will be transferred to a password protected, nominated user restricted access REDCap database that will be housed on a secure server at the UoL. This server will be backed up and maintained in line with institutional compliance regulations for Data Protection and Information Security.

The study coordinator will consistently check that all study documentation stored in the CfWHR is pseudonymised and identifiable only by a unique study identification number (except for signed consent forms, which will be stored in a separate locked cabinet in the CfWHR).

The Chief Investigator will preserve the confidentiality of participants taking part in the study and will abide by the EU General Data Protection Regulation 2016 and Data Protection Act 2018.

**7.4 QUALITY ASSURANCE AND QUALITY CONTROL OF DATA**

Systems of quality assurance, including all elements described in this protocol have been/ will be implemented within relevant institutions with responsibility for this study. QC is applied to each stage of data handling to ensure that data are accurate, reliable and processed correctly.

The study site, facilities, laboratories and all data (including sources) and documentation must be available for GCP audit and inspection by competent authorities or IEC. Such audits / inspections may take place at any site where study related activity is taking place (the Sponsor’s site(s), the CfWHR or at any investigator’s site including laboratories).

The site staff should assist in all aspects of audit/inspection and be fully cognisant of the Sponsor communication strategy for single and multicentre studies. This includes management systems for the green light process prior to participant recruitment at site.

**7.5 RECORDS RETENTION**

The investigator at LWH site must make arrangements to store the essential study documents, (as defined in Essential Documents for the Conduct of a Clinical Study (ICH E6 (R2), Guideline for Good Clinical Practice)) including the Investigator Study File, until the Sponsor or the CfWHR informs the investigator that the documents are no longer to be retained.

In addition, the investigator is responsible for archiving of all relevant source documents so that the study data can be compared against source data after completion of the study (e.g. in case of inspection from authorities). The investigator is required to ensure the continued storage of the documents, even if the investigator, for example, leaves the clinic/practice or retires before the end of the required storage period. Delegation must be documented in writing. The CfWHR undertakes to store all electronic data related to completed database, except for source documents pertaining to the individual investigational site, which are kept by the investigator only. At the point where it is decided that the study documentation is no longer required; the Investigator will be responsible for the destruction of all site study specific documentation and the Sponsor /CfWHR will be responsible for the destruction of all study related materials retained.

Verification of appropriate informed consent will be enabled by the provision of copies of participants signed ICF being supplied to the CfWHR by LWH (recruiting site). This requires that name data will be transferred to the CfWHR, which is explained in the PIS. The CfWHR will preserve the confidentiality of participants taking part in the study and the UoL is a Data Controller registered with the Information Commissioners Office.

**7.6 STUDY DISCONTINUATION**

If this study is prematurely discontinued (e.g., due to safety) all participants must be informed and the reason for the discontinuation should be written on the end of study form for each participant. If a participant has been withdrawn completely from the study whilst the study is still ongoing, an end of study form should be completed.

**7.7 INDEMNITY**

The University of Liverpool professional indemnity and clinical trials insurance will apply as appropriate.

**7.8 SPONSOR**

The University of Liverpool.

**7.9 FUNDING**

Funded through the Harris-Wellbeing Preterm Birth Centre and the Centre for Women’s Health Research. Pregnolia AG provided the device for use in this study and provided financial aid to fund a Clinical Research Fellow to undertake the research.

Pregnolia AG and the study sponsor had no role in the study design and will not be involved in study management, data collection or analysis or subsequent publication.

**7.10 AUDITS**

The study may be subject to inspection and audit by regulatory bodies to ensure adherence to GCP and the UK Policy Framework for Health and Social Care Research (v3.2 10th October 2017).

**8. STUDY MANAGEMENT**

The day‐to‐day management of the study will be coordinated through the Centre for Women’s Health Research, Department of Women’ and Children’s Health.

**8.1 STUDY MANAGEMENT GROUP (SMG)**

A Study Management Group will be formed comprising the CI, other lead investigators / core study management staff who are central to the day-to-day running of the study. The SMG will be responsible for the day-to-day running and management of the study and will meet at regular intervals throughout the course of the study. The frequency of meetings will be decided by the CI. However it is expected that they should take place at least monthly. This group may consist of the following members of the core research team however all are not required to attend each meeting in person. Compulsory attendance in denoted by a *.

- *Chief Investigator
- Research Manager
- *Co-applicants
- Sponsor representative

**8.2 OVERSIGHT COMMITTEES**

This study will not convene both a Steering Committee or an Independent Safety and Data Monitoring Committee (ISDMC) as this is a low risk non-interventional prospective cohort study. Oversight will be provided by the SMG and Sponsor.

**9. END OF STUDY**

The study will end when the last recruited woman has delivered and both herself and her baby have been discharged from hospital, or 1 month after delivery, whichever is sooner and all planned analysis of collected data has taken place.

**10. MONITORING**

Central and study centre monitoring is conducted to ensure protection of participants in the study, and that procedures and data collection processes are of high quality and meet sponsor and, when appropriate, regulatory requirements. A risk assessment will be carried out to determine the level of monitoring required, and a subsequent monitoring plan will be developed to document who will conduct the central (and potentially site) monitoring, at what frequency monitoring will be carried out and the level of detail at which monitoring will be conducted.

A full quality control check of the protocol has been completed by the Centre for Women’s Health Research and the Chief Investigators. In addition, a SPIRIT (Standard Protocol Items: Recommendations for Interventional Trials) check has also been carried out in line with standard guidelines.

**10.1 RISK ASSESSMENT**

In accordance with the requirements of the Sponsor, a risk assessment has been completed in partnership with:

- Representatives of the Study Sponsors (University of Liverpool)
- Chief Investigator
- Members of the Study Management Group

In conducting this risk assessment, the contributors considered potential patient, organisational and study hazards, the likelihood of their occurrence and resulting impact should they occur. The outcome of the risk assessment is categorised based upon the potential risk associated with the study intervention in accordance with MRC/DH/MHRA Project on Risk-adapted Approaches to the Management of Clinical Trials:

<http://www.mhra.gov.uk/home/groups/l-ctu/documents/websiteresources/con111784.pdf>

- Type A: No higher than that of standard medical care
- Type B: Somewhat higher than that of standard medical care
- Type C: Markedly higher than that of standard medical care
- Non-CTIMP

**The initial risk assessment for this study resulted in a study category of Low Risk. This is a non-CTIMP.**

**10.2 SOURCE DATA**

Source data are all information, original records of clinical findings, observations, or other activities in a clinical study necessary for the reconstruction and evaluation of the study. Source data are contained in source documents (original records or certified copies). (ICH E6 (R2), 1.51).

**10.2.1 Source documents**

Original documents and data records include: hospital records, clinical and office charts, laboratory notes, memoranda, subjects’ diaries or evaluation checklists, pharmacy dispensing records, recorded data from automated instruments, copies or transcriptions certified after verification as being accurate and complete, microfiches, photographic negatives, microfilm or magnetic media, x-rays, subject files, and records kept at the pharmacy and laboratory departments involved in the clinical study. (ICH E6 (R2). 1.52)

For data where no prior record exists and which are recorded directly in the study workbooks/CRFs (e.g., vital signs) the study workbooks/CRFs will be considered the source document unless otherwise indicated by the investigator.

All data recorded in the workbooks/CRFs should be consistent and verifiable with source data in source documents other than the workbook (e.g., medical record, laboratory reports and medical notes, scan reports). For this reason, the study centre should maintain appropriate medical and research records for this study in compliance with ICH E6 (R2) GCP, Section 4.9 and regulatory and institutional requirements for the protection of confidentiality of study participants for the purpose of source data verification.

**10.3 DATA CAPTURE METHODS**

Study data will be initially captured using paper case report forms (CRFs). The data from the paper CRFs will then be transcribed to an electronic CRF (eCRF) within a bespoke study database. This database is designed and maintained by the UoL in collaboration with the Chief Investigator and Study Co-Ordinator. The eCRF is the primary data collection instrument for the study. All data requested on the eCRF must be recorded and all missing data explained.

**10.3.1 Case Report Forms**

All paper CRFs will be transcribed onto the bespoke study database, accessed via a secure webpage by members of the CASPAR research team. The client application is secured with a unique username / password combination allocated to each delegated member of the research team. When data is entered into the study database it is electronically stamped with the date, time and the person who entered it. If data is changed, it is electronically stamped with the change and will be accompanied with the date, time, person and a reason for making the change or correction. The previous value is recorded in an audit trail for each data item.

The study database contains specific validation checks on the data being entered. If any values are outside what is expected, or data are missing, this is flagged up and will be raised as a discrepancy on the main database system. Regular reports will be generated to identify discrepancies in the data, and allow for follow up. Comprehensive guidelines for data entry will be provided to all staff who have been delegated the responsibility for data collection. After data has been transcribed from the CRF (within 24 hours of study visit) it will be stored in a locked filing cabinet, in a locked room with restricted access and key card access within the CfWHR, UoL.

Paper screening logs will be kept to record the number of patients declining participation and when volunteered the reason given. This data will also be kept in a secure locked location in the CfWHR, UoL.

Paper workbooks/CRFs will be available from the CASPAR Study Co-Ordinator. To ensure current versions of the workbook/CRF are used, QC processes including on site source data verification will be put in place in line with the study database and all up to date workbooks/CRFs should be kept in the ISF at the research site (LWH).

**10.4 MONITORING METHODS**

There are a number of monitoring features in place to ensure reliability and validity of the study data.

**10.4.1 Green Light Process**

The Green Light Process ensures that all regulatory and ethical approvals are in place, contracts / agreements are signed and study management standard operating procedures are in place prior to the study opening. Furthermore, a site greenlight process is will also be followed that ensures all study specific and ICH GCP training has been completed for site research staff before a study site is open and able to register participants. The green light process for opening this study will be managed by the Centre for Women’s Health Research.

**10.4.2 Site Research Staff**

All site research staff involved in the study must be included on the delegation log. The PI at site (LWH) signs off on the delegation log only those staff members he/she feels are able and competent to complete the assigned tasks. The delegation log provides clearly defined delegation of responsibility thus ensuring site research staff are aware of their responsibilities, and is continuously checked against staff named on CRFs, SAE reports and registration forms.

The Study Co-Ordinator/CI will ensure that as a minimum the PI, a research nurse, and a member of laboratory staff at site have study-specific training (on the protocol, SAE reporting and consent process) all of which is provided at site initiation (either on site or by teleconference) by the Study Co-Ordinator/CI. The PI is responsible for ensuring site staff named on the delegation log but not present at site initiation receive study-specific training (on the protocol, SAE reporting and consent process). LWH will be provided with copies of training aids presented at site initiation to provide a constant reminder of key issues.

Delegated site research staff must also submit their CV and provide the date of their last ICH GCP training. In order to ensure that site research staff maintain up to date ICH GCP training (to be renewed every 3 years as agreed by the Sponsor). An automated email reminder will be sent to site research staff when their next ICH GCP training is due. Non-NHS staff must have honorary NHS contracts and evidence of CRB checks must be obtained for staff (when necessary by UK law).

**10.4.3 Oversight**

The CASPAR study will convene a regular Study Management Group (SMG). A formal Oversight Committee is not required for this study as it is a prospective observational study with minimal risk involved to study participants. Data collection and quality processes are in place and will be managed centrally by the CfWHR.

**10.4.4 Safety Reporting**

Safety reports will be generated by the Study Co-Ordinator every 3 months, which allow monitoring of SAE reporting rates. Any concerns raised by the SMG or inconsistencies noted may prompt additional training, with the potential for the Study Co-Ordinator to carry out site visits if there is suspicion of unreported SAEs in participant case notes. Additional training will also be provided if unacceptable delay in safety reporting timelines is noted. AE’s will be reviewed regularly by the SMG.

**10.4.5 Eligibility and Consent**

The Study Co-Ordinator will verify that all site research staff attended study-specific training relating to eligibility screening and the informed consent/registration process. The Study Co-Ordinator will carry out a check of all consent forms sent to the CfWHR. This includes checking that the patient is eligible, the correct versions of the PIS and ICF have been used and the patient and clinician signatures are present and dated on the same day.

**10.4.6 Participant Confidentiality**

All study management and site research staff have received ICH GCP training and are thus aware of the importance of patient confidentiality. The Study Co-Ordinator will consistently check that all study documentation sent to the CfWHR are pseudonymised and identifiable only by a unique study identification number (except for signed consent forms, which are stored in a separate locked cabinet in the CfWHR). The Study Co-Ordinator will monitor site performance on maintaining patient confidentiality and will provide additional training if a particular site sends any patient identifiers to the CfWHR (other than on the signed consent form).

**10.4.7 Recruitment**

The Study Co-Ordinator will produce regular recruitment reports, to allow the SMG to review recruitment. Slow or inconsistent recruitment will trigger further action centrally. The Study Co-Ordinator may liaise directly with site staff in order to query reasons for slow recruitment and try to resolve any problems that could impact recruitment. The Study Co-Ordinator will check that the study is being actively promoted at the research site (LWH), and site recruitment schedules will be reviewed during the course of the study as necessary.

**10.4.8 Protocol Violations/Deviations**

All protocol violations and deviations will be recorded by the Study Co-Ordinator in the study site status database and are included in regular reports. The Study Co-Ordinator will send details of all protocol violations and deviations to the CI as soon as they have been made aware of them. The CI will then consider whether any are potential serious breaches that would need to be forwarded immediately to the Sponsor. If it is noted that a particular site is making consistent protocol violations or deviations, additional training will be provided by the Study Co-Ordinator.

**10.4.9 Withdrawals/ Losses to Follow Up and Missing Data**

The Study Co-Ordinator will produce reports on withdrawals, losses to follow-up and the quantity of missing CRF data across sites for review by the SMG. Identified problems will be discussed and remedial action taken as necessary.

As outlined in the data management plan, the Study Co-Ordinator will check that withdrawal CRFs are completed for all withdrawn participants (including the reasons for withdrawal). The Study Co-Ordinator will review withdrawal rates and reasons for withdrawal, paying particular attention to withdrawals close to dates of registration. If LWH experiences an excessive rate of withdrawals, additional training on the informed consent procedure will be provided.

**10.4.10 Data Management Plan**

All CRF data entered into the bespoke study database will be centrally monitored by the CfWHR to ensure that data collected are consistent with adherence to the study protocol. The bespoke database used for this study includes validation features which will alert the user to certain inconsistent or missing data on data entry. If any problems are identified via automated validation or central monitoring, a query is raised and emailed to site (LWH). A complete log of discrepancies and data amendments is automatically generated, including the date of each change, the reason for the change and the person who made the change, thus providing a complete audit trail.

Additional site training will be carried out if recurring problems are noted with data, such as consistently incorrect or incomplete data, a backlog of unresolved queries, or unacceptable time delays in submitting CRFs. This study will have a separate Data Management Plan, which will detail all components of data management for this study.

**10.4.11 Centre for Women’s Health Research Staff**

All CfWHR study management staff will receive regular ICH GCP training, have in-house training records and undergo regular individual Performance Development Review (PDR) sessions, all of which are used to ensure that appropriate training is received and any problems identified and resolved in a timely fashion.

**10.4.12 Statistical Monitoring**

All statistical analyses will be carried out using licensed software, with evidence of completed validation testing performed at regular intervals. Statistical coding and subsequent results relating to study analyses will be checked and saved in the study folder. Confirmation of this will be evidenced in the Study Management Folder.

**10.5 CLINICAL SITE MONITORING**

**10.5.1 Direct Access to data**

If necessary, a study monitor may need direct access to primary participant data, e.g. participant records, laboratory reports, appointment books, etc. Each PI therefore permits study related monitoring, audits, ethics committee review and regulatory inspections by providing direct access to source data/documents. As this affects the participant’s confidentiality, this fact is included on the Participant Information Sheet and ICF.

**10.5.2 Confidentiality**

Individual participant medical information obtained as a result of this study is considered confidential and disclosure to third parties is prohibited. CRFs will be labelled with participant unique study identification numbers. Consent forms sent to the CfWHR as part of the registration process may contain patient identifiers for the purpose of monitoring as described in the study risk assessment. Such information will be stored in secure, locked cabinets and participants will be asked to explicitly consent to this transfer.

**10.5.3 Quality Assurance and Quality Control of data**

See Section 7.4.

**10.5.4 Records Retention**

See Section 7.5.

**11. ARCHIVING**

Data and all appropriate documentation will be stored for a minimum of 15 years after the completion of the study, including the follow‐up period, unless otherwise directed by the funder/sponsor/regulatory bodies. All data will be processed in line with the CfWHR, and Sponsor archiving procedures and stored in the CfWHR, UoL. At the point where it is decided that the study documentation is no longer required; the Investigator will be responsible for the destruction of all site study specific documentation and the Sponsor/HWPBC will be responsible for the destruction of all study related materials retained.

**12. PUBLICATION POLICY**

The results of this study will be published once all study data has been collected, validated and analysed. Individual researchers must undertake not to submit any part of their individual data for publication without the prior consent of the SMG.

The SMG will form the basis of the Writing Committee and advise on the nature of publications. The Uniform Requirements for Manuscripts Submitted to Biomedical Journals (http://www.icmje.org/) will be respected. All publications shall include a list of participants, and if there are named authors, these should include the study’s CI(s), Statistician(s) and Study Manager(s) involved at least. If there are no named authors (i.e. group authorship) then a writing committee will be identified that would usually include these people, at least.

The members of the Study Oversight Committees should be listed with their affiliations in the Acknowledgements / Appendix of the main publication. All publications arising from the CASPAR Study will be open access.

**13. REFERENCES**-

1. (NICE) NIfHaCE. Induction of Labour (CG70) 2021 [Available from: <https://www.nice.org.uk/guidance/ng207/evidence/full-guideline-july-2008-pdf-9266823757>.

2. NHS Maternity Statistics E. 2020-21 [Available from: <https://digital.nhs.uk/data-and-information/publications/statistical/nhs-maternity-statistics/2020-21>.

3. Artuso H, Davis DL. Trends and characteristics of women undergoing induction of labour in a tertiary hospital setting: A cross-sectional study. Women Birth. 2022;35(2):e181-e7.

4. Declercq E, Belanoff C, Iverson R. Maternal perceptions of the experience of attempted labor induction and medically elective inductions: analysis of survey results from listening to mothers in California. BMC Pregnancy Childbirth. 2020;20(1):458.

5. Marconi A. Recent advances in the induction of labor [version 1; peer review: 2 approved]. F1000Research [Internet]. 2019. Available from: <https://doi.org/10.12688/f1000research.17587.1>.

6. Middleton P, Shepherd E, Crowther CA. Induction of labour for improving birth outcomes for women at or beyond term. Cochrane Database Syst Rev. 2018;5:CD004945.

7. Meier K, Parrish J, D'Souza R. Prediction models for determining the success of labor induction: A systematic review. Acta Obstet Gynecol Scand. 2019;98(9):1100-12.

8. D'Souza R, Ashraf R, Foroutan F. Prediction models for determining the success of labour induction: A systematic review and critical analysis. Best Pract Res Clin Obstet Gynaecol. 2022;79:42-54.

9. Ezebialu IU, Eke AC, Eleje GU, Nwachukwu CE. Methods for assessing pre-induction cervical ripening. Cochrane Database Syst Rev. 2015(6):CD010762.

10. Bishop EH. Pelvic Scoring for Elective Induction. Obstet Gynecol. 1964;24:266-8.

11. Burnett JE, Jr. Preinduction scoring: an objective approach to induction of labor. Obstet Gynecol. 1966;28(4):479-83.

12. Baacke KA, Edwards RK. Preinduction cervical assessment. Clin Obstet Gynecol. 2006;49(3):564-72.

13. Badir S, Bernardi L, Feijo Delgado F, Quack Loetscher K, Hebisch G, Hoesli I. Aspiration technique-based device is more reliable in cervical stiffness assessment than digital palpation. BMC Pregnancy Childbirth. 2020;20(1):391.

14. Abdullah ZHA, Chew KT, Velayudham VRV, Yahaya Z, Jamil AAM, Abu MA, et al. Pre-induction cervical assessment using transvaginal ultrasound versus Bishops cervical scoring as predictors of successful induction of labour in term pregnancies: A hospital-based comparative clinical trial. PLoS One. 2022;17(1):e0262387.

15. Hendrix NW, Chauhan SP, Morrison JC, Magann EF, Martin JN, Jr., Devoe LD. Bishop score: a poor diagnostic test to predict failed induction versus vaginal delivery. South Med J. 1998;91(3):248-52.

16. Kolkman DG, Verhoeven CJ, Brinkhorst SJ, van der Post JA, Pajkrt E, Opmeer BC, et al. The Bishop score as a predictor of labor induction success: a systematic review. Am J Perinatol. 2013;30(8):625-30.

17. Eggebo TM, Okland I, Heien C, Gjessing LK, Romundstad P, Salvesen KA. Can ultrasound measurements replace digitally assessed elements of the Bishop score? Acta Obstet Gynecol Scand. 2009;88(3):325-31.

18. Rane SM, Guirgis RR, Higgins B, Nicolaides KH. The value of ultrasound in the prediction of successful induction of labor. Ultrasound Obstet Gynecol. 2004;24(5):538-49.

19. Migliorelli F, Rueda C, Angeles MA, Banos N, Posadas DE, Gratacos E, et al. Cervical consistency index and risk of Cesarean delivery after induction of labor at term. Ultrasound Obstet Gynecol. 2019;53(6):798-803.

20. Londero AP, Schmitz R, Bertozzi S, Driul L, Fruscalzo A. Diagnostic accuracy of cervical elastography in predicting labor induction success: a systematic review and meta-analysis. J Perinat Med. 2016;44(2):167-78.

21. Parra-Saavedra M, Gomez L, Barrero A, Parra G, Vergara F, Navarro E. Prediction of preterm birth using the cervical consistency index. Ultrasound Obstet Gynecol. 2011;38(1):44-51.

22. Banos N, Murillo-Bravo C, Julia C, Migliorelli F, Perez-Moreno A, Rios J, et al. Mid-trimester sonographic cervical consistency index to predict spontaneous preterm birth in a low-risk population. Ultrasound Obstet Gynecol. 2018;51(5):629-36.

23. Badir S, Mazza E, Zimmermann R, Bajka M. Cervical softening occurs early in pregnancy: characterization of cervical stiffness in 100 healthy women using the aspiration technique. Prenat Diagn. 2013;33(8):737-41.

24. Badir S, Mazza E, Bajka M. Objective Assessment of Cervical Stiffness after Administration of Misoprostol for Intrauterine Contraceptive Insertion. Ultrasound Int Open. 2016;2(2):E63-7.

25. Teresi JA, Yu X, Stewart AL, Hays RD. Guidelines for Designing and Evaluating Feasibility Pilot Studies. Med Care. 2022;60(1):95-103.

26. Dos Santos F, Drymiotou S, Antequera Martin A, Mol BW, Gale C, Devane D, et al. Development of a core outcome set for trials on induction of labour: an international multistakeholder Delphi study. BJOG. 2018;125(13):1673-80.

27. Son M. Core outcome set for induction of labour trials: what's the expectation? BJOG. 2018;125(13):1681.

28. Alavifard S, Meier K, Shulman Y, Tomlinson G, D'Souza R. Derivation and validation of a model predicting the likelihood of vaginal birth following labour induction. BMC Pregnancy Childbirth. 2019;19(1):130.

**14. APPENDICES**

**14.1 REQUIRED DOCUMENTATION**

CASPAR Consent form

CASPAR Patient information sheet

CASPAR Post-assessment patient questionnaire

**14.2 SCHEDULE OF STUDY PROCEDURES**

| **Procedure** | | **Induction of labour Booking** | **Attendance for induction of labour** | **Postnatal** | **End of Study** |
| --- | --- | --- | --- | --- | --- |
| *Patient Eligibility* | | **X** | **X** |  |  |
| *Patient information Leaflet provided* | | **X** | **X** |  |  |
| *Informed Consent* | | **X** | **X** |  |  |
| *Routine Assessments* | *USS for fetal presentation* |  | **X** |  |  |
|  | *CTG* |  | **X** |  |  |
| *Cervical Stiffness assessment* | |  | **X** |  |  |
| *Bishop’s score assessment* | |  | **X** |  |  |
| *Post- assessment patient questionnaire* | |  | **X** |  |  |
| *Primary Outcome* | |  |  | **X** |  |
| *Secondary outcomes* | |  |  | **X** |  |
| *Adverse Event Reporting* | |  | **x** | **x** | **x** |
